# Orthopædia

**Published:** 1897-11

**Authors:** Frank W. Sage


### Orthopædia.

BY FRANK W. SAGE.

Read before the Cincinnati Odontological Society, May, 1897.

In the outset of preparing this paper, I found so much that relates more particularly to orthodontia to interest me, that I regret now that I did not announce my subject in that word. What I really want to talk about is orthodentia—a word not approved by Dr. Farrar—incidentally adverting to orthopædia later.

A glance over the collected writings of the few men who are recognized more particularly as specialists in this phase of dental art, reveals some interesting features of disagreement on matters of no little importance to one seeking for the best modes and the best appliances for correcting dental irregularities. For instance, Dr. Farrar questions whether any considerable space is gained, ever, by widening the arch, that is, space enough to admit of outstanding teeth being drawn into line. Claims that many so-called successes are really failures—"*ridiculous failures*"—to use his words. Are we to infer from this that Dr. Farrar approves of enlarging the arch only as a means of improving facial expression? He advocates extracting the first bicuspid in crowded conditions of incisors and cuspids, arguing that if the second bicuspid be taken out, one more tooth remains to be moved back, and moreover, the advantage of having a bicuspid for anchorage, is lost. I have to suggest in passing, two other reasons, not as yet noticed, to my knowledge, by writers on this subject, for sacrificing the first in preference to the second bicuspid. The first reason is, in the many cases where we wish to draw the cuspid down and back to make room for crowded incisors, the cuspid may be drawn down bodily into the vacated socket of the bicuspid, in an incredibly short time, and with ease. This is an advantage which will be readily appreciated by all who consider the great difficulty, ordinarily, of moving these teeth at all.

The other reason for retaining the second in preference to the first bicuspid, is, that in the event later, of a pivoted crown being required for the second bicuspid, its root better favors the operation than does that of the first bicuspid.

Dr. Jackson discourages extraction in correcting irregularities. He enlarges the arch by means of piano wire springs soldered to stays resting against the teeth. He disapproved of jack-screws, which are an important adjunct of both Farrar's and Angle's systems. Dr. Matteson (Chicago), has difficulty in so manipulating piano wire in the mouth, as to get the direction of force and the degree of force which he requires. To which the writer says, "same here." Bending it or kinking it a little with pliers means that youv'e changed its shape without knowing at all how you have altered its use as a lever. Jackson argues that the screw is unintermittent. But we may give our patients' teeth a rest by judiciously abstaining from twisting the screw. Objection overruled—in the writer's opinion.

Among other things worth remembering, is the concurrence of several writers in the opinion that it is never necessary to open the bite in order to allow of inlocked lateral incisors being forced into line. As soon as the teeth begin to be sore, the patient will open his mouth enough to allow them to pass forward. To which the writer, as the result of personal observation and experience, also subscribes.

A comparison of various methods and appliances for regulating teeth is of advantage in enabling us to decide what is best to employ in our practice. He who sets about carefully to study various *systems* for regulating teeth, has this advantage over the inventors of the systems—he is not hampered by personal pride or personal prejudice in either adopting or rejecting any good idea. One man disapproves of screws possibly because he didn't suggest their use in the first place. Another objects to wire springs for the same reason. But we, who are interested only in the question, how best to regulate our patients' teeth, will use whichever we find most agreeable to our patients, most convenient for ourselves, most effective in accomplishing the end sought. Thus we find, for instance, that the Jackson curved spring for widening arches, lying up out of the way, is more agreeable to our patients than a jack-screw, but on the whole we would prefer Angle's cemented bands with Jackson's spring wire, because of the greater stability of the bands over Jackson's crib attachment.

So much that looks practical and effective on paper, fails in the mouth. Thus Angle's claim that a stiff band firmly cemented to a molar and having in turn a lever firmly soldered to it—for retracting a cuspid, we will say—may be depended on to resist any tendency in the molar to tip. But in actual use this claim is disproved: the molar tips. Dr. Angle it will be noticed, bands two or three contiguous teeth when practicable, as if distrustful of his own suggestion.

Finally, as to orthopedia: Has there been an over-enthusiasm as regards the possibility of improving facial appearance and expression by moving the bones and the associated four incisor teeth? We can conceive of some classes of faces where such changes about the mouth as Dr. Case proposes and actually brings about, would not result in improvement. First; in idiots. Even in faces various degrees removed above idiocy, faces merely weak, silly, characterless, the result of remodeling the mouth might be disappointing; in fact, in the writer's office, adverse criticism has been made of so-claimed improvement—as shown by photographs —of two of Dr. Case's remodeled faces.

In the cases of mouth deformity where the slope of the face is very marked, whether it be a receeding of the lower part of the face or of the upper part, no material improvement should be looked for from remodeling the mouth, chin, or the alar regions of the nose. Given a feminine face with strongly masculine features; large, prominent, aggressive; no possible modification of a single feature could materially alter the expression of the individual's face. The same statement is true of a masculine face with small, feminine features; no remodeling of a part will materially change the expression of the whole. You may raise a depressed part of a weak face, with the effect, not of strengthening the expression, but of rendering it grotesque. Take from a strong, mishapen face some one of its prominent features, and you may find that you have wrought a heightened effect of disharmony and incongruity.

These suggestions are offered merely by way of admonition against indiscriminately resorting to means for accomplishing an end commendable in many cases, but worse than useless in many others.

